# Circulating microparticles and central blood pressure according to antihypertensive strategy

**DOI:** 10.6061/clinics/2019/e1234

**Published:** 2019-11-04

**Authors:** Nayara D. Massunaga, Carolina N. França, Henrique T. Bianco, Carlos E.S. Ferreira, Juliana T. Kato, Rui M.S. Póvoa, Antonio M. Figueiredo Neto, Maria Cristina O. Izar, Francisco Antonio Helfenstein Fonseca

**Affiliations:** I Departamento de Medicina, Universidade Federal de Sao Paulo (UNIFESP), Sao Paulo, SP, BR; II Universidade Santo Amaro (UNISA), Sao Paulo, SP, BR; III Hospital Israelita Albert Einstein, Sao Paulo, SP, BR; IV Instituto Nacional de Ciencia e Tecnologia de Fluidos Complexos, Universidade Sao Paulo, Sao Paulo, SP, BR

**Keywords:** Endothelial Microparticles, Monocyte Microparticles, Platelet Microparticles, Central Blood Pressure, Hydrochlorothiazide, Amlodipine

## Abstract

**OBJECTIVES:**

This prospective, randomized, open-label study aimed to compare the effects of antihypertensive treatment based on amlodipine or hydrochlorothiazide on the circulating microparticles and central blood pressure values of hypertensive patients.

**METHODS:**

The effects of treatments on circulating microparticles were assessed during monotherapy and after the consecutive addition of valsartan and rosuvastatin followed by the withdrawal of rosuvastatin. Each treatment period lasted for 30 days. Central blood pressure and pulse wave velocity were measured at the end of each period. Endothelial, monocyte, and platelet circulating microparticles were determined by flow cytometry. Central blood pressure values and pulse wave velocity were recorded at the end of each treatment period.

**RESULTS:**

No differences in brachial blood pressure were observed between the treatment groups throughout the study. Although similar central blood pressure values were observed during monotherapy, lower systolic and diastolic central blood pressure values and early and late blood pressure peaks were observed in the amlodipine arm after the addition of valsartan alone or combined with rosuvastatin. Hydrochlorothiazide-based therapy was associated with a lower number of endothelial microparticles throughout the study, whereas a higher number of platelet microparticles was observed after rosuvastatin withdrawal in the amlodipine arm.

**CONCLUSIONS:**

Despite similar brachial blood pressure values between groups throughout the study, exposure to amlodipine was associated with lower central blood pressure values after combination with valsartan, indicating a beneficial interaction. Differences between circulating microparticles were modest and were mainly influenced by rosuvastatin withdrawal in the amlodipine arm.

## INTRODUCTION

Arterial hypertension is responsible for high morbidity and mortality rates around the world. After the publication of the 2017 American College of Cardiology (ACC)/American Heart Association (AHA) blood pressure guidelines, a substantial increase in the number of hypertensive adults eligible for drug therapy is expected (1).

Hypertension is a progressive and complex disease that is associated with anatomical and functional changes in blood vessels, such as endothelial dysfunction, inflammation, and disturbance in adaptive and innate immunity (2-4). Indeed, antihypertensive drugs have been widely used to achieve appropriate blood pressure levels and prevent comorbidities (5-8).

Blood pressure monitoring is essential for the treatment of hypertension. However, the assessment of peripheral blood pressure may be insufficient to achieve better control of this complex disease. Thus, the measurement of vascular compliance and central blood pressure can provide important information to expand the possibilities of clinical treatment (9-11).

In addition to these measurements, endothelium-, platelet-, and monocyte-derived microparticles have been proposed as novel vascular biomarkers because they have been related to endothelial erosion, thrombosis, and atherosclerosis (12-14). Some differences in microparticle concentrations have been described in relation to the use or withdrawal of statins (15). Thus, considering the high cardiovascular risk of many hypertensive individuals, the analysis of the concomitant use of statins, even in patients with adequate blood pressure control, seems relevant.

In the ACCOMPLISH study (16), subjects with similar blood pressure control received combined antihypertensives based on two different strategies, and the difference in cardiovascular outcomes highlighted the relevance of the effects of these drugs on vascular biology (17,18). Furthermore, central blood pressure responses to therapy may differ from peripheral blood pressure responses, thus explaining some differences in cardiovascular outcomes, as described in the Conduit Artery Function Evaluation (CAFE) study (19,20).

The present study aimed to compare the effects of an antihypertensive treatment based on amlodipine or hydrochlorothiazide on circulating endothelium-, monocyte-, and platelet-derived microparticles and central blood pressure parameters. Thus, new aspects of antihypertensive therapy that might be related to different clinical outcomes are described here, as reported in the ASCOT and ACCOMPLISH studies.

## MATERIALS AND METHODS

### Ethics

This prospective, randomized, open-label trial with blinded endpoints complied with the Declaration of Helsinki and was approved by the Ethics Committee of UNIFESP. Patients were invited to participate and were included in the trial after they agreed to the study protocol and signed a written informed consent form.

### Study population

Adult hypertensive patients (aged 40-75 years, of both sexes) were enrolled if they had systolic blood pressure (SBP) or diastolic blood pressure (DBP) in the ranges of 160-179 and/or 100-119 mmHg, respectively, without pharmacological treatment; or 140-159 and/or 90-99 mmHg, respectively, or appropriate blood pressure levels (<140 and/or <90 mm Hg) with the use of two antihypertensive drugs. The key exclusion criteria were as follows: secondary hypertension, prior coronary heart disease or stroke, estimated glomerular filtration rate less than 30 mL/min per 1.73 m^2^ of body-surface area, history of neoplasm in the last five years, or active liver disease.

**Figure 1:**
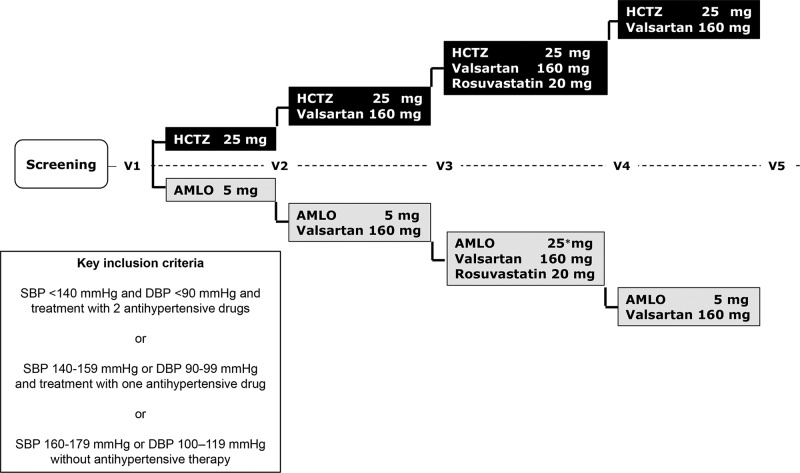
Study design. Eligible hypertensive subjects were randomized to receive amlodipine (5 mg daily) or hydrochlorothiazide (25 mg daily) for 4 weeks (V1), followed by three consecutive 4-week periods of treatment with the addition of valsartan (V2) and rosuvastatin (V3) and the withdrawal of rosuvastatin (V4) in both study arms. Blood samples for measurement of circulating microparticle concentrations and central blood pressure levels were obtained at the end of each treatment period. The use of antihypertensive and lipid-lowering drugs was suspended in V1 when monotherapy with either hydrochlorothiazide or amlodipine was initiated.

The trial was designed to compare the effects of antihypertensive treatments (strategy: 30 successive days) based on hydrochlorothiazide (Clorana^®^; Sanofi Aventis Pharma, 25 mg daily) or amlodipine (Amlocor^®^, Torrent Pharma, 5 mg daily) on circulating microparticles. At baseline, all subjects received nutritional counseling, and those using lipid-lowering drugs suspended their use. The first period of monotherapy (hydrochlorothiazide or amlodipine) was followed by the addition of valsartan (Brasart^®^, Torrent Pharma; 160 mg daily) and rosuvastatin (Rosucor^®^, Torrent Pharma; 20 mg daily), and the withdrawal of rosuvastatin with the maintenance of the antihypertensive drugs was performed in the last 30 days of treatment. Central blood pressure and pulse wave velocity (PWV) were also measured at the end of each 30-day treatment (Figure 1).

### Circulating microparticles

Circulating endothelium-, monocyte- and platelet-derived microparticles were measured as previously reported (21,22). In brief, blood samples were collected and centrifuged (160g; 20-22°C; 10 min) to obtain platelet-rich plasma (PRP). The PRP was centrifuged (1500g; 20-22°C; 6 min) to obtain platelet-poor plasma (PPP). Furthermore, PPP (50 µL) was labeled (20 min; room temperature) with CD51FITC, CD42FITC and CD31 PE, and CD14FITC (BD Biosciences) for the identification of endothelial, platelet, and monocytic microparticles, respectively. Isotypes (BD Biosciences) were used as controls. Microparticles were quantified per microliter of PPP injected into the cytometer, according to the standard protocol. TruCOUNT (BD Biosciences) tubes containing a known number of beads were used to quantify the number of microparticles per microliter of PPP.

### Pulse wave velocity and peripheral and central blood pressure

Ambulatory blood pressure, central blood pressure, and PWV were recorded for 24h at the end of each treatment period using a Dyna-MAPA Plus (Cardios, Brazil) (23).

### Statistical Analysis

Data are expressed as the mean (± standard deviation) or median (and interquartile range) for continuous variables, and n (%) is used for categorical variables. The Kolmogorov-Smirnov test was used to evaluate normality. Continuous variables that were normally distributed were evaluated by ANOVA-GLM. Microparticles were compared (within group and between groups) using the Friedman and Mann-Whitney (nonparametric) tests, respectively. SPSS software (v. 18.0) was used for statistical analysis. A significance level of an alpha risk less than 5% was adopted for all tests.

## RESULTS

### Population

Female, elderly, and overweight individuals (n=46) were more prevalent in the sample. Table 1 shows the major baseline characteristics of the study population. The pharmacological therapies were well tolerated, and no serious adverse events were recorded in either group. Both groups had similar blood pressure values at baseline and after 4 weeks of monotherapy with amlodipine (AMLO) or hydrochlorothiazide (HCTZ) (Table 2).

### Biochemical parameters

No changes in glucose or creatinine serum levels were observed throughout the study. After a 4-week exposure to rosuvastatin (V4), a significant and similar decrease was observed in serum levels of total cholesterol and LDL-C in both groups (*p*<0.0001 *vs*. V2, V3, and V5) (Table 3).

### Brachial blood pressure

Brachial blood pressure was measured in both groups (AMLO and HCTZ) and showed similar values for SBP and DBP at each medical visit. However, higher values were observed at V2 in both arms. Differences within and between groups can be calculated from the data shown in Table 2.

### Central blood pressure

Central blood pressure was monitored for 24h from V2 to V5 in eight patients in the AMLO and HCTZ groups. The mean values obtained were similar to those obtained at V2. In the HCTZ arm, no difference between medical visits was observed in the central SBP or DBP values throughout the study. However, lower central blood pressure was recorded beginning at V3 in the AMLO arm. PWV values did not change during the study, and similar results were recorded in both groups at each visit from V2 to V5. Central blood pressure values are shown in Table 4.

### Circulating microparticles

**Figure 2:**
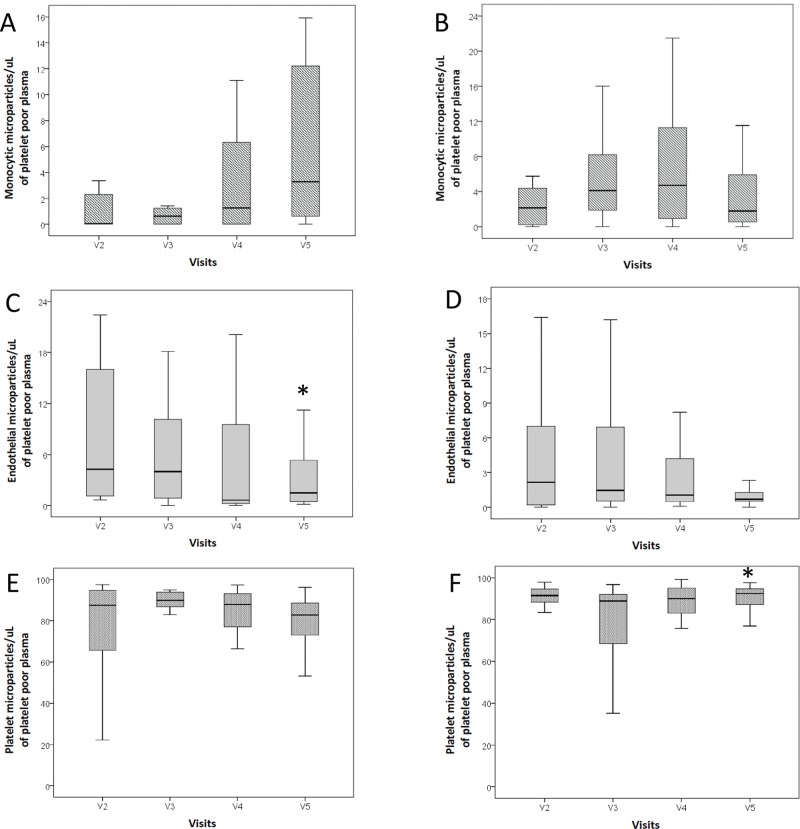
Box plots of microparticle concentrations by treatment period (visits, V) and study arm. *indicates significant differences. Monocytic (A), endothelial (B), and platelet (C) microparticle concentrations after the hydrochlorothiazide-based treatment. Monocytic (D), endothelial (E), and platelet (F) microparticle concentrations after the amlodipine-based treatment. The concentration of endothelial microparticles decreased after exposure to hydrochlorothiazide (*p*=0.001; Friedman test). The concentration of platelet microparticles increased after withdrawal of rosuvastatin in the amlodipine-based arm (*p*=0.045; Friedman test).

The AMLO-based treatment did not change plasma concentrations of monocyte-derived microparticles (MMP) or endothelium-derived microparticles (EMP) throughout the study. However, more platelet-derived microparticles (PMP) were observed at visit 5 after rosuvastatin withdrawal (Friedman test; *p*=0.045). Regarding the HCTZ-based treatment, no difference was observed in the amount of circulating PMP or MMP throughout the study, except for a lower number of EMP at V5 (Friedman test; *p*=0.001). Higher levels of MMP (visit 2) and PMP (visit 5) were observed in the AMLO group (Mann-Whitney test; *p*=0.011 and *p*=0.003, respectively). Table 5 shows the number of circulating microparticles at each visit and differences between treatment groups. Figure 2 shows the effects of amlodipine- or hydrochlorothiazide-based therapies on the circulating levels of microparticles throughout the study.

## DISCUSSION

The study revealed that amlodipine- or hydrochlorothiazide-based antihypertensive therapies may have different effects on circulating microparticles. Furthermore, although central blood pressure values were similar during monotherapy, differences in central blood pressure parameters were noted after the addition of valsartan and/or rosuvastatin (not detected by brachial blood pressure measurements).

Circulating microparticles, also called extracellular vesicles or shedding vesicles, are released from the endothelium, platelets, and many other cells and may have physiological or pathological effects (24). The diameters of circulating microparticles are usually in the range of 100-500 nm, and an elevated number of circulating microparticles has been reported in many pathological conditions and in subjects with uncontrolled hypertension (25), acute myocardial infarction (26), or chronic kidney disease (27).

The first interesting finding of the study was related to MMP. During monotherapy, the four-week exposure to amlodipine was associated with higher levels of MMP than those associated with hydrochlorothiazide exposure, although in both groups, the brachial blood pressure values were higher than the recommended value range, which was expected because the majority of participants in both arms were previously treated by two antihypertensive agents. Elevated MMP values were previously reported in hypertensive subjects with type 2 diabetes, particularly among those with high SBP (28). In addition, the higher titers of MMP in the amlodipine arm may be related to harmful effects on endothelial cells because MMP contains interleukin 1-beta (IL-1β), a cytokine that activates the inflammatory cascade related to cardiovascular disease (29,30). Hydrochlorothiazide decreases blood pressure but not the levels of high-sensitivity C-reactive protein, even when combined with valsartan, an angiotensin II blocker (ARB) with anti-inflammatory properties (31). Interestingly, hydrochlorothiazide seems to neutralize the benefits of renin-angiotensin system blockade in the context of experimental atherosclerosis (32). The amounts of circulating EMP were similar in both antihypertensive therapy arms, but lower levels were observed throughout the study in the HCTZ group. Conversely, a marginal increase in the levels of PMP was observed in the AMLO group after discontinuation of rosuvastatin, even without a change in the values of central or brachial arterial blood pressures. A similar finding was observed after discontinuation of rosuvastatin in subjects with stable coronary heart disease (14), with an increase in the amount of PMP despite the continuous use of antiplatelet therapy. Interestingly, not only blood pressure control but also lipid-lowering therapy may influence the amount of circulating PMP. Overall, the effects of amlodipine and hydrochlorothiazide on circulating microparticles seem to be affected differently by concomitant therapies.

Regarding central blood pressure, our study also showed differences between the AMLO- and HCTZ-based therapies, with lower SBP and DBP values in the AMLO-based arm. Indeed, these differences became apparent only after the addition of valsartan, suggesting a beneficial interaction between amlodipine and valsartan, an effect not observed with hydrochlorothiazide. A recent study showed that despite similar brachial blood pressure values, ARBs were more effective than atenolol for reducing central blood pressure, indicating their potential benefit for the prevention of cerebrovascular events (33). Furthermore, a meta-analysis examining the effects of combined therapies on brachial and central blood pressures showed that betablockers and diuretics were less effective in reducing central blood pressure (34). However, the combined treatment with chlorthalidone and amiloride was effective in reducing central blood pressure, suggesting possible differences in effectiveness between diuretics in terms of vascular benefits (35,36).

Aortic augmentation index (AIx) has been recognized as an indicator of arterial stiffness and wave reflection. Changes in these parameters and PWV may require long-term exposure to therapies and seem to be related to the choice of antihypertensive therapy (37). In fact, a meta-analysis exploring the effects of antihypertensives on AIx and PWV revealed lower AIx values associated with ARBs compared to the effects of other antihypertensives. However, the authors failed to demonstrate a lesser decrease in PWV, probably due to other concomitant medications, comorbidities, age, and time of drug exposure (38). In our study, the exposure to combined therapies was only 12 weeks, which was possibly insufficient for changes involving vascular remodeling. Differences in AIx values were not observed in either group, but the wave pressures were lower in the AMLO group after the addition of valsartan.

### Limitations

Our prospective randomized study should be considered a pilot study. First, we enrolled a relatively small number of patients and examined the effects of therapies on short-term outcomes. In addition, we were unable to compare the effects of the treatments with baseline visits (V1) due to the need to homogenize treatments via the withdrawal of antihypertensives and statins. However, after 4 weeks of monotherapy with amlodipine or hydrochlorothiazide, the central and brachial blood pressures were similar, and changes in circulating microparticles and central blood pressure were observed shortly after the addition of ARB or ARB/statin. These findings may contribute to explaining some intriguing differences in cardiovascular outcomes in trials of combined therapies, such as the ACCOMPLISH study, despite similar brachial blood pressure values achieved, especially among diabetic patients (39).

Our observations are promising, although they are preliminary and require confirmation in larger prospective studies. Exploratory analysis of central parameters and PWV suggest that new technologies, which allow for the registry of central blood pressure, vascular compliance, and other hemodynamic data, are essential for establishing new targets beyond brachial blood pressure for the long-term treatment of patients with cardiovascular hypertensive disease.

## CONCLUSIONS

The amlodipine- or hydrochlorothiazide-based antihypertensive therapy had different effects on circulating microparticles and central blood pressure parameters. Despite similar brachial arterial blood pressures and biochemical parameters throughout the study, exposure to amlodipine was associated with lower central blood pressure values after combination with valsartan, indicating a beneficial interaction. Differences in the amount of circulating microparticles were modest but were mainly influenced by rosuvastatin withdrawal in the amlodipine group.

## AUTHOR CONTRIBUTIONS

Massunaga ND and França CN collected clinical data, performed laboratory tests, performed statistical analyses, and drafted the manuscript. Bianco HT and Ferreira CES performed screening, conducted the medical visits and contributed important intellectual content. Kato JT and Póvoa RMS performed blood pressure monitoring and contributed with important critical interpretations of the collected data. Figueiredo Neto AM and Izar MCO performed manuscript revision and provided financial support. Fonseca FAH designed the study and critically reviewed the manuscript.

## Figures and Tables

**Table 1:** Baseline characteristics of the study population receiving treatment based on amlodipine (AMLO) or hydrochlorothiazide (HCTZ).

Characteristics (mean±SE unless otherwise stated)	HCTZ (n=22)	AMLO (n=24)
Age (years)*	63 (56-69)	66 (60-71)
Female sex**	21 (95)	21 (87)
Diabetes**	9 (39)	11 (46)
BMI (kg/m^2^)	29.3±1.1	32.1±1.4
SBP (mm Hg)	133±2	133±2
DBP (mm Hg)	81±2	78±2
Antihypertensives prior to randomization**		
None	2 (9)	1 (4)
One	4 (18)	6 (25)
Two	16 (73)	17 (70)
Lipid-lowering therapy (statins)	14 (64)	19 (79)

* median (interquartile range);

** n (%); n: number; SE: standard error; BMI: body-mass index; SBP: systolic blood pressure; DBP: diastolic blood pressure; LDL-C: low-density lipoprotein cholesterol; HDL-C: high-density lipoprotein cholesterol. No difference was observed between groups regarding baseline characteristics (unpaired *t* or Pearson’s chi-square test).

**Table 2:** Brachial systolic and diastolic blood pressure levels (mmHg; median and interquartile range) as measured at the visits after treatment with hydrochlorothiazide (HCTZ, 25 mg) or amlodipine (AMLO, 5 mg).

Visits	Brachial BP	HCTZ N=22	AMLO N=24	*p-*values
V_1_	sBP	131 (127-138)	132 (126-139)	0.89
	dBP	78 (69-87)	81 (75-88)	0.35
V_2_	sBP	143 (133-151)	142 (133-149)	0.59
	dBP	81 (75-80)	85 (80-91)	0.21
V_3_	sBP	121 (115-127)	127 (113-139)	0.22
	dBP	75 (71-80)	74 (68-80)	0.43
V_4_	sBP	123 (119-130)	122 (113-130)	0.88
	dBP	74 (69-79)	73 (70-78)	0.65
V_5_	sBP	122 (114-130)	126 (117-134)	0.40
	dBP	75 (70-79)	77 (73-80)	0.40

V_1_: screening; V_2_: monotherapy: hydrochlorothiazide or amlodipine; V_3_: valsartan, 160 mg, added in both arms; V_4_: rosuvastatin, 20 mg, added in both arms; V_5_: rosuvastatin was withdrawn in both arms. sBP: systolic blood pressure; dBP: diastolic blood pressure. The Mann-Whitney U test was used for comparisons.

**Table 3:** Biochemical characteristics (mean ± standard error) by visit and treatment based on hydrochlorothiazide (HCTZ, 25 mg) and amlodipine (AMLO, 5 mg).

Parameters (mg/dL)	Visits	HCTZ (N=22)	AMLO (N=24)	*p-*values*
Glycemia	V_2_	110 (6)	121 (9)	0.75
	V_3_	118 (13)	106 (9)	0.42
	V_4_	108 (14)	116 (13)	0.71
	V_5_	104 (13)	114 (9)	0.28
Creatinine	V_2_	0.61 (0.03)	0.65 (0.03)	0.36
	V_3_	0.67 (0.04)	0.60 (0.04)	0.27
	V_4_	0.63 (0.06)	0.62 (0.05)	0.85
	V_5_	0.62 (0.06)	0.57 (0.05)	0.78
Cholesterol	V_2_	270 (19)	232 (11)	0.79
	V_3_	233 (20)	217 (12)	0.46
	V_4_	145 (14)	145 (9)	0.99
	V_5_	220 (24)	219 (17)	0.74
LDL-C	V_2_	123 (23)	137 (12)	0.21
	V_3_	127 (17)	129 (10)	0.91
	V_4_	78 (9)	67 (7)	0.37
	V_5_	133 (17)	132 (14)	0.81
HDL-C	V_2_	48 (2)	57 (3)	0.77
	V_3_	46 (2)	53 (4)	0.22
	V_4_	42 (4)	51 (4)	0.10
	V_5_	41 (4)	49 (4)	0.07
Triglycerides	V_2_	287 (72)	198 (59)	0.67
	V_3_	207 (30)	162 (31)	0.33
	V_4_	129 (19)	130 (25)	0.99
	V_5_	247 (47)	199 (70)	0.69

* Between groups. V_2_ (monotherapy: hydrochlorothiazide or amlodipine); V_3_ (valsartan, 160 mg added in both arms); V_4_ (rosuvastatin, 20 mg, added in both arms); V_5_ (rosuvastatin withdrawal in both arms).

**Table 4:** Central blood pressure levels (mmHg; median and interquartile range) and pulse wave velocity (PWV, m/s; median and interquartile range) by visit and treatment based on hydrochlorothiazide (HCTZ, 25 mg) and amlodipine (AMLO, 5 mg).

Visits	Parameters	HCTZ (n=8)	AMLO (n=8)	*p*-values
V_2_	HR	73 (69-71)	74 (70-78)	0.833
	cSBP	109 (106-118)	116 (109-131)	0.430
	cDBP	76 (72-81)	73 (69-81)	0.461
	AIx75	31 (23-35)	37 (31-39)	0.102
	CO	3.9 (3.6-4.2)	3.9 (3.8-4.1)	0.959
	P_1_	101.5 (94.7-104.9)	98.9 (93.2-111.1)	0.916
	P_2_	109.7 (105.5-117.8)	115.8 (109.9-131.9)	0.401
	PWV	8.77 (8.13-9.98)	9.94 (7.93-10.25)	0.600
V_3_	HR	78 (68-92)	74 (69-85)	0.923
	cSBP	118 (110-119)	105 (100-111)	0.012*
	cDBP	75 (72-82)	64 (61-80)	0.074
	AIx75	32.2 (26.7-37.5)	36.6 (28.4-41.9)	0.370
	CO	4.12 (3.78-4.55)	3.62 (3.34-4.55)	0.167
	P_1_	105.9 (98.2-106.6)	90.6 (84.3-99.1)	0.027*
	P_2_	118.1 (109.9-118.5)	105.1 (99.6-111.1)	0.012*
	PWV	9.07 (8.38-10.05)	9.77 (7.85-9.98)	0.700
V_4_	HR	72 (71-81)	68 (67-85)	0.757
	cSBP	112 (105-118)	103 (97-106)	0.009*
	cDBP	73 (72-73)	66 (58-75)	0.009*
	AIx75	29.8 (21.8-41.6)	36.3 (29.3-39.8)	0.200
	CO	3.82 (3.57-4.27)	3.41 (3.17-3.89)	0.094
	P_1_	96.9 (94.9-102.7)	88.4 (81.5-94.0)	0.002*
	P_2_	112.4 (104.7-118.2)	102.9 (97.5-106.1)	0.009*
	PWV	8.98 (8.33-11.21)	9.57 (7.63-10.03)	0.825
V_5_	HR	75 (69-81)	72 (66-74)	0.101
	cSBP	111 (109-120)	107 (98-111)	0.023*
	cDBP	74 (73-78)	64 (58-77)	0.039*
	AIx75	35.1 (23.4-35.7)	36.7 (29.7-37.3)	0.791
	CO	4.07 (3.98-4.64)	3.41 (3.31-3.70)	0.016*
	P_1_	100.1 (96.7-103.9)	91.0 (81.0-96.6)	0.010*
	P_2_	111.1 (108.6-120.5)	106.9 (98.1-111.2)	0.023*
	PWV	8.38 (6.57-8.85)	9.72 (7.84-10.05)	0.266

HR: heart rate (beats/min); cSBP: central systolic blood pressure; cDBP: central diastolic blood pressure; AIx75: heart rate adjusted augmentation index; CO: cardiac output; P_1_: early blood pressure peak; P_2_: late blood pressure peak. V_2_ (monotherapy: hydrochlorothiazide or amlodipine); V_3_ (valsartan, 160 mg, added in both arms); V_4_ (rosuvastatin, 20 mg, added in both arms); V_5_ (rosuvastatin withdrawal in both arms).

* Significant differences between arms (Mann-Whitney’s U test).

**Table 5:** Levels of circulating microparticles (MPs; mg/dL; median and interquartile range) by visit and treatment based on hydrochlorothiazide (HCTZ, 25 mg) or amlodipine (AMLO, 5 mg).

Visits	MPs	HCTZ N=22	AMLO N=24	*p-*values
V_2_	MMP	0.00 (0.00-1.71)	2.14 (0.21-4.55)	0.011*
	EMP	1.63 (1.11-11.91)	2.09 (0.20-6.46)	0.097
	PMP	93.8 (82.0-95.8)	91.7 (88.5-94.7)	0.403
V_3_	MMP	0.70 (0.00-1.34)	4.11 (1.84-8.48)	0.088
	EMP	3.87 (0.79-6.20)	1.37 (0.53-6.16)	0.131
	PMP	92.4 (87.1-94.5)	88.9 (71.6-92.7)	0.466
V_4_	MMP	0.86 (0.00-8.26)	4.71 (0.92-11.73)	0.316
	EMP	0.39 (0.22-0.69)	1.03 (0.48-4.18)	0.481
	PMP	88.1 (74.7-94.1)	90.2 (83.5-95.4)	0.451
V_5_	MMP	3.35 (0.43-12.5)	1.79 (0.27-6.17)	0.125
	EMP	1.02 (0.40-4.38)	0.69 (0.43-1.27)	0.200
	PMP	85.8 (75.0-90.4)	92.9 (87.3-94.9)	0.003*

MMP: monocytic microparticles; EMP: endothelial microparticles; PMP: platelet microparticles. V_1_ (at screening); V_2_ (monotherapy: hydrochlorothiazide or amlodipine); V_3_ (valsartan, 160 mg, added in both arms); V_4_ (rosuvastatin, 20 mg, added in both arms); V_5_ (rosuvastatin withdrawal in both arms).

* Higher number of circulating microparticles in the AMLO group (Mann-Whitney’s U test).
